# Cyclin-Dependent Kinase Inhibitors Function as Potential Immune Regulators *via* Inducing Pyroptosis in Triple Negative Breast Cancer

**DOI:** 10.3389/fonc.2022.820696

**Published:** 2022-06-08

**Authors:** Tao Xu, Zhen Wang, Jiahao Liu, Ge Wang, Dongchen Zhou, Yaying Du, Xingrui Li, Yu Xia, Qinglei Gao

**Affiliations:** ^1^ Cancer Biology Research Center (Key Laboratory of the Ministry of Education), Tongji Hospital, Tongji Medical College, Huazhong University of Science and Technology, Wuhan, China; ^2^ Department of Obstetrics and Gynecology, Tongji Hospital, Tongji Medical College, Huazhong University of Science and Technology, Wuhan, China; ^3^ Department of Thyroid and Breast Surgery, Tongji Hospital, Tongji Medical College, Huazhong University of Science and Technology, Wuhan, China

**Keywords:** triple negative breast cancer, GW-8510, CDK inhibitor, pyroptosis, connectivity map database

## Abstract

**Background:**

Immunotherapy is the most promising treatment in triple-negative breast cancer (TNBC), and its efficiency is largely dependent on the intra-tumoral immune cells infiltrations. Thus, novel ways to assist immunotherapy by increasing immune cell infiltrations were highly desirable.

**Methods:**

To find key immune-related genes and discover novel immune-evoking molecules, gene expression profiles of TNBC were downloaded from Gene Expression Omnibus (GEO). Single-sample gene set enrichment analysis (ssGSEA) and Weighted Gene Co-expression Network Analysis (WGCNA) were conducted to identified hub genes. The CMap database was used subsequently to predicate potential drugs that can modulate the overall hub gene expression network. *In vitro* experiments were conducted to assess the anti-tumor activity and the pyroptosis phenotypes induced by GW-8510.

**Results:**

Gene expression profiles of 198 TNBC patients were downloaded from GEO dataset GSE76124, and ssGSEA was used to divide them into Immune Cell Proficiency (ICP) group and Immune Cell Deficiency (ICD) group. Hub differential expressed gene modules between two groups were identified by WGCNA and then annotated by Gene Ontology (GO) annotation and Kyoto Encyclopedia of Genes and Genomes (KEGG) pathway enrichment analysis. A cyclin-dependent kinase (CDK) 2 inhibitor, GW-8510 was then identified by the CMap database and further investigated. Treatment with GW-8510 resulted in potent inhibition of TNBC cell lines. More importantly, *in vitro* and *in vivo* studies confirmed that GW-8510 and other CDK inhibitors (Dinaciclib, and Palbociclib) can induce pyroptosis by activating caspase-3 and GSDME, which might be the mechanism for their immune regulation potentials.

**Conclusion:**

GW-8510, as well as other CDK inhibitors, might serve as potential immune regulators and pyroptosis promotors in TNBC.

## Introduction

Representing 15% of breast cancers, Triple Negative Breast Cancer (TNBC) is the most aggressive breast malignancy (1, 2). The absence of hormone receptors and human epidermal growth factor receptor 2 (HER2) makes TNBC not respond to targeted therapies and exhibit a poor prognosis (3). Chemotherapy, although with relatively high clinical response rates, its clinical application is limited by unavoidable toxicities and growing prevalence of chemoresistance (4, 5). Thus, emerging novel efficient treatments such as immunotherapy are becoming highly desirable in TNBC (6).

In the past decades, tremendous efforts have been made to restore antitumor immunity. Among them, the most famous one is the broad application of immune checkpoint therapy, which prevent the effective T cells from dysfunction and normalized their anti-tumor activities, thus preventing immune escape, which is a hallmark of carcinogenesis and a major cause of cancer metastasis and progression, and ensuring prolonged remissions eventually (7–9). Despite the encouraging results achieved in various malignancies such as non-small-cell lung cancer and melanoma, the response rate of immune checkpoint therapy in TNBC is far from satisfaction (10). Favorable responses to immunotherapy are observed only in a small subset of TNBC patients (11), with an overall response rate ranged between 5% and 20% across different trials (5). What’s more, compares to chemotherapy, pembrolizumab monotherapy did not produce significant long-term survival benefits (12). Therefore, it is important to find new ways to boost the immune treatment efficiency and ensure an advantageous long-term prognosis in patients with TNBC.

According to previous research, the efficiency of immunotherapies is largely dependent on the intra-tumoral immune cell infiltrations (13, 14). Recent studies have segregated the tumor immune microenvironment into three main phenotypes, namely “the immune-desert phenotype”, “the immune–excluded phenotype” and “the inflamed phenotype” (15). And robust anti-PD-1 efficiency was established in the presence of pre-treatment tumor-infiltrating T lymphocytes, with immune-infiltrated tumors achieving better responses than immune-desert ones (16). The recruitment of peripheral T cells into tumor microenvironments has also been recognized as the functional fundamental for Immune Checkpoint Blocker- (ICB-) induced anti-tumor activities (17, 18). Thus, strategies to increase intra-tumoral immune cell infiltrations may assist anti-cancer immunotherapy.

Previous studies have revealed several ways to increase immune cell infiltrations, including modulating immune-related gene expressions. However, considering the complicity of immune system, the perturbation of single genes might have limited power in reshaping the entire tumor immune microenvironment (13). Therefore, we hypothesized that the global modification of genes whose expressions are related to immune cell infiltrations in TNBC may be more efficient. Small molecular agents have been reported to play an active role in modulating gene clusters globally in previous studies (19, 20), this study was thus conducted to identify and verify drugs with the potential to increase immune cell infiltration *via* the perturbation of related genes.

In this study, hub genes related to immune cell infiltrations in TNBC were revealed using comprehensive bioinformatics analysis. The tight associations among hub genes enabled them to be regulated globally by GW-8510, an inhibitor of cyclin-dependent kinase (CDK) 2. What’s more, the anti-tumor activity and the ability to induce pyroptosis of CDK inhibitors were validated for the first time, and the latter is likely to be the major mechanism for increased immune cell infiltration.

## Methods

### Gene Expression Data Acquisition and Patient Classification

The gene expression profiles and clinical information of 198 TNBC patients were downloaded from Gene Expression Omnibus (GEO, accession number: GSE76124) (21, 22).

TNBC samples were grouped by single-sample gene set enrichment analysis (ssGSEA) according to the gene expression signatures of immune cell types and immune pathway enrichment (23). An immune cell proficiency (ICP) and an immune cell deficiency (ICD) group were established accordingly. Distinct immune microenvironments between the two groups were further revealed by significant differences in Stromal Score, Immune Score, ESTIMATE Score, Tumor Purity Score, and the immune cell infiltration fractions calculated by the CIBERSORT algorithm. The above analysis was conducted on R software using the R package “GSVA” and “hclust”.

### Gene Co-Expression Network Construction

Weighted Gene Co-expression Network Analysis (WGCNA) was performed to assess the co-expression similarities among genes and their correlations with immune cell infiltration. Genes with similar expression peculiarities established the same module. Gene significance (GS, the correlation between the gene and the immune cell infiltration) and module membership (MM, the correlation between the gene and the gene modules) were used to quantify the configurations of modules and features. WGCNA was performed using R software (version 3.6.2).

### Functional Enrichment and Protein-Protein Interaction Analysis

The Gene Ontology (GO) annotation and Kyoto Encyclopedia of Genes and Genomes (KEGG) pathway enrichment analysis for targeted genes were accomplished by “clusterProfiler” and “enrichplot” on R software. STRING (http://www.string-db.org/) database was used to calculate the associations among selected genes and construct protein-protein interaction (PPI) networks.

### Connectivity Map Analysis

By documenting gene expression perturbations after pharmacologic interfering, the CMap database (https://www.broadinstitute.org/drug-repurposing-hub) can recommend compounds based on the given gene expression changes (24). In this study, hub gene expression differences between ICP and ICD groups were uploaded for the compound prediction. The enrichment scores are calculated (between 0 to 1), and molecules with enrichment scores close to 1 are supposed to be able to enhance the query gene expression pattern and have therapeutic potentials.

### Cell Culture and Reagents

Mouse mammary carcinoma cell line (4T1), and human TNBC cell lines (BT549, MDA-MB-231) were purchased from ATCC. MDA-MB-231 were maintained in Dulbecco’s modified Eagle’s medium (DMEM, Gibco) with 10% fetal bovine serum, 1‰ penicillin/streptomycin. 4T1 and BT549 were maintained in RPMI-1640 Medium (Gibco) with 10% fetal bovine serum, 1‰ penicillin/streptomycin. All cell lines were incubated at 37°C in a humidified incubator with 5% CO2. GW-8510 (CAS 222036-17-1) was pursued from Santa Cruz Biotechnology, Inc. Dinaciclib (HY-10492), and Palbociclib (HY-50767) were pursued from MedChemExpress.

### Cytotoxic Assay

BT549, MDA-MB-231, and 4T1 cells were seeded in 96-well plates at a density of 2×10^3^ cells per well and incubated overnight at 37°C with 5% CO2. Cells were then treated **with different concentrations** of GW-8510 or DMSO (vehicle control) **for 24 h, 48 h, or 72 h**, followed by Cell Counting Kit- (CCK-) 8 for another 2 h at 37°C. The absorbance at 450 nm was measured using a Varioskan Flash microplate reader (Thermo Scientific, Waltham, MA, United States).

### Colony Formation Assay

A total of 1×10^3^ cells per well were seeded evenly into six-well plates and incubated at 37°C overnight. After treatment with gradient concentrations (0, 1.25, and 2.5 µM) of GW-8510 for 24 hours. The medium was discharged and cells were cultured with fresh medium for another 10 days. Then, cells were washed with pre-warmed PBS, fixed with 4% PFA, and stained with Giemsa solution for 15 min.

### LDH Release Assay

To determine the LDH release caused by GW-8510, BT549, MDA-MB-231, and 4T1 cells were seeded in 12-well plates and incubated grown to almost 50%-60%. Cells were then treated with different concentrations (0, 2.5, 5, and 10 µM) of GW-8510 or DMSO (vehicle control) for 24 h. Then the medium was collected and the LDH release was detected using Cytotoxicity Detection Kit (LDH) (11644793001, Sigma-Aldrich^®^ Brand) according to the manufacturer’s instructions.

### Flow Cytometry Assay

For evaluation of apoptosis, cells were treated with gradient concentrations of GW-8510 for 24 h, and then labeled with the Annexin V-FITC Apoptosis Detection Kit (BD Biosciences, USA) following the manufacturer’s protocol. Thereafter, cells were analyzed immediately using a flow cytometry FACS Calibur System (Beckman Coulter).

### Western Blot Analysis

Cells were washed with ice-cold PBS and lysed in RIPA lysis buffer with protease inhibitors on ice for 20 min, followed by centrifugation at 13,000 rpm for 30 min at 4°C, and the supernatants were collected. Protein concentrations were then determined and 20 µg total protein was resolved in 10% SDS-PAGE gels followed by electrophoretic transfer onto PVDF membrane. Blots were blocked at room temperature for 1 h in 5% BSA Tris-buffered saline (TBS)–Tween (TBS-T) on a shaker and then incubated with the primary antibodies overnight at 4°C. The membrane was washed in TBS-T and then incubated with horseradish peroxidase (HRP)-conjugated anti-rabbit or anti-mouse immunoglobulin G at room temperature for 1 h. Immunoreactive proteins were then detected by ECL reagent according to the manufacturer’s protocol. And the following antibodies were used: anti-GAPDH (1:2,000; catalog: 10494-1-AP; Proteintech), anti-Cleaved Caspase-3 (1:1,000; catalog: ab32042; Abcam), and anti-DFNA5/GSDME-N-terminal (1:1,000; catalog: ab215191; Abcam).

### Xenograft Study

4-6-week-old female BALB/c mice were purchased from Beijing HFK Bioscience Co. Ltd. The mice were housed in a specific pathogen-free (SPF) environment at Laboratory Animal Care Center of Tongji Hospital, and allowed to recover and were monitored closely for one week before any treatment. Then 4T1 breast cancer cells (1×105) were subcutaneously injected into the right posterior limb. For treatment, mice were randomized into two groups (n=5 per group), vehicle and Dinaciclib, since tumor volumes reaches 50 mm3. Mice were treated 3 times per week with Dinaciclib 30mg/kg administered *via* i.p injection. The tumor size was monitored every 3 days. Tumor length and width were measured using electronic calipers. The tumor volume was calculated as follows: volume = 0.5 × length × width2. At sacrifice, portions of tumors were stored in liquid nitrogen for follow-up western blot test or were fixed in 4% Polyformaldehyde for routine histopathologic processing. And the following antibodies were used: anti- HMGB1 (1:400; catalog: ab79823; Abcam), anti-CD8 (1:2,000; catalog: ab209775; Abcam), and anti-Granzyme B (1:3,000; catalog: ab255598; Abcam). All animal procedures were performed in accordance with the approved Guide for the Care and Treatment of Laboratory Animals of Tongji Hospital and approved by the Ethics Committees of Tongji Hospital.

### Statistical Analysis

Data were expressed as the means ± standard deviation. The WGCNA method was analyzed by Pearson correlation analysis. Statistical analyses were performed using Student’s t-test or one-way analysis of variance (ANOVA) followed by a *post-hoc* test. *P < 0.05, **P < 0.01, and ***P < 0.001 were considered statistically significant.

## Results

### Establishment of Immune Groups

**Figure 1:**
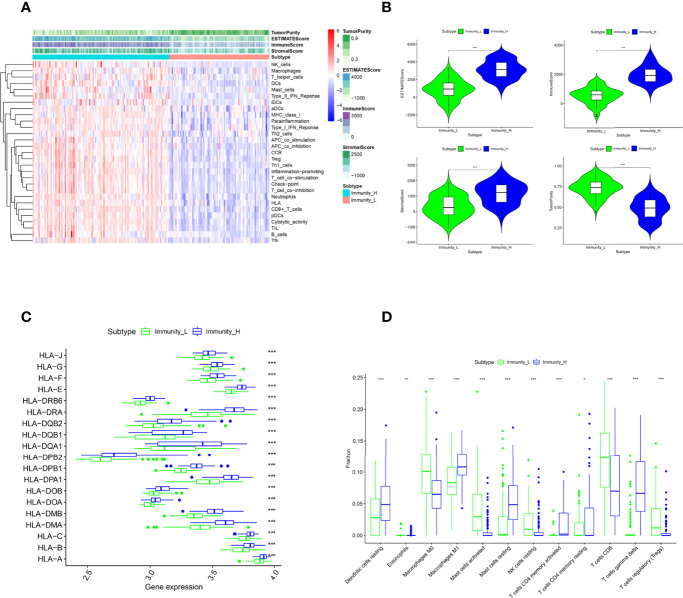
Patient classification by immune cell infiltration. **(A)**. The ssGSEA divided patients into Immune Cell Proficiency (ICP) group and Immune Cell Deficiency **(ICD)** group. The Tumor Purity, ESTIMATE Score, Immune Score, and Stromal Score of each sample gene were also displayed. **(B)**. The difference in Tumor Purity, ESTIMATE Score, Immune Score, and Stromal Score between the two groups. **(C)** The expression of HLA family genes in the two groups. **(D)** The immune cell infiltration fractions in the two groups caculated by the CIBERSORT algorithm. *P < 0.05, **P < 0.01, and ***P < 0.001 were considered statistically significant.

198 TNBC samples were obtained from Gene Expression Omnibus (GEO) and then grouped as the immune cell proficiency (ICP) (n = 114) or immune cell deficiency (ICD) (n = 84) group by single-sample gene set enrichment analysis (ssGSEA) (**Figure 1A**). Samples of the ICP group had enriched genes signatures in immune cell infiltrations (including NK cells, Macrophages, T helper cells, Dendritic cells, Mast cells, Treg cells, neutrophils, CD8+ T cells, and B cells) and immune pathways (Type I/II Interferon responses, Antigen presentation activities, Inflammations, T cell co-stimulation\inhibitions, and Cytolytic activities). Distinct immune microenvironments were established in two groups, evidenced by lower Tumor Purity but higher ESTIMATE Score, Immune Score, and Stromal Score in the ICP group (**Figure 1B**). Higher expression of HLA genes can also be observed in the ICP group (**Figure 1C**), along with the increased fraction of M1 macrophage, Dendritic cells, and CD4+ T memory cells calculated by CIBERSORT algorithm (**Figure 1D**). Interestingly, T gamma/delta cells, which have been revealed as the most favorable prognostic T cells (25), were also significantly increased in the ICP group.

### Identification of Genes Related to High Immune Cell Infiltration by WGCNA

**Figure 2:**
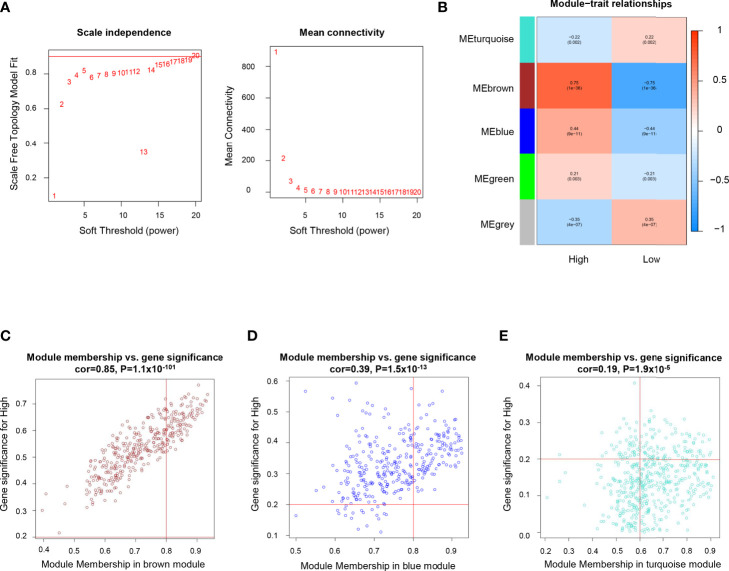
Identification of genes related to high immune cell infiltration. **(A)** Determination of soft threshold by evaluating the scale-free topology fit index (left) and mean connectivity (right). **(B)** Heatmap for the correlations of gene modules to immune cell infiltration. Correlations between module membership and gene significance values were presented in scatterplots for the brown **(C)** blue **(D)**, and turquoise **(E)** gene modules.

Differentially expressed gene modules between ICP and ICD group were identified by Weighted Gene Co-expression Network Analysis (WGCNA). The soft threshold β value equaled 15 to satisfy the scale-free topology for the co-expression network (**Figure 2A**). Five gene modules were identified (**Figure 2B**) and labeled with different colors (turquoise, brown, blue, green, and grey). Genes within the blue and brown modules were more likely to be overexpressed in the ICP group, while the upregulated gene expressions in the turquoise and grey module were more commonly seen in the ICD group (**Figure 2B**).

In the immune positive-related gene modules (brown and blue module), the hub genes referred to those with module membership (MM) >0.8 and gene significance (GS) >0.2. As a result, 98 genes in the brown modules (**Figure 2C**), and 108 genes in the blue modules (**Figure 2D**) were revealed as the hub genes and included for further analysis. While in the grey and turquoise module, which is negatively related to immune infiltration, only 78 genes in the turquoise module with MM >0.6 and GS >0.2 were considered to be immune negative-related hub genes (**Figure 2E**).

### Annotation of Hub Genes

The Gene Ontology (GO) annotation and Kyoto Encyclopedia of Genes and Genomes (KEGG) analysis were then conducted to annotate the biological activity related to hub genes. For immune positive-related hub genes (genes in the brown and blue modules), the GO analyses revealed that the dominant biological functions included T cell and lymphocyte activation (**Figure S1A**). Both KEGG and GO analysis displayed that these hub genes took an active part in the biological process of cell adhesion (**Figures S1A, B**). In KEGG analysis, pathways concerning immune cell regulation (leukocyte transendothelial migration, natural killer cell-mediated cytotoxicity, Th1/Th2 cell differentiation, hematopoietic cell lineage, Th17 cell differentiation, and T cell receptor signaling pathway) were revealed to be closely related to the hub genes (**Figure S1B**). For genes negatively related to immune infiltration, the most involved biological process disclosed by KEGG and GO analysis was cell cycle (cell cycle checkpoint and the regulation of mitotic cell cycle phase transition in GO; cell cycle in KEGG) and cell division (nuclear division, organelle fission, chromosome segregation, and mitotic nuclear division in GO; DNA replication and Oocyte meiosis in KEGG) (**Figures S2A, B**).

### PPI Network and Profiling of Co-Expressed Genes

**Figure 3:**
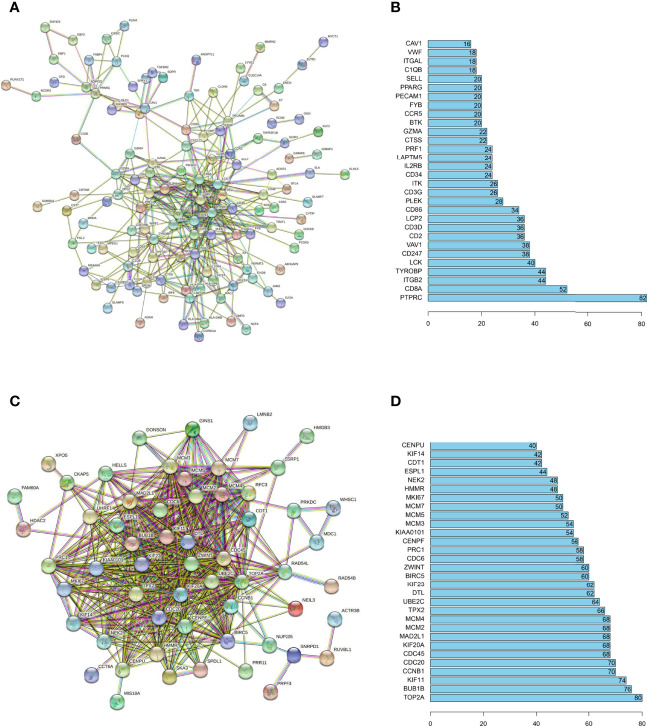
PPI networks analysis for immune related hub genes. PPI networks were drawn by STRING (URL: https://string-db.org/) for hub proteins positive- **(A)** and negative- **(C)** related to immune infiltration. The minimum interaction score was set at 0.7. The disconnected nodes were not shown in the network. The number of connections was also listed for hub proteins of the immune positive- **(B)** and negative- **(D)** related hub genes.

The inherent associations among hub genes were demonstrated on the transcriptomic and proteomic levels using co-expression coefficients and protein-protein interaction (PPI)networks to see if these genes could be modulated globally. The strong intercorrelations for gene expressions within the immune positive- and negative- related gene modules were demonstrated and attested by the correlation plot (**Figures S3A, B** **)**. Similar conclusions could be drawn on the protein level (**Figure 3**). In the brown and blue (immune positively related) modules, key immune regulatory molecules including PTPRC (26, 27) (also known as CD45, the key molecular in TCR activation and lymphocyte proliferation), CD8a (28, 29) (a glycoprotein that defines cytotoxic effector cells), LCK (30, 31) (a key signaling molecule in the selection and maturation of developing T-cells), and CD247 (32, 33) (also known as CD3, presented on the T-lymphocyte cell surface that played an essential role in adaptive immune response) seemed to be the center of PPI network, with interaction number of 82, 52, 40 and 38 respectively (**Figures 3A, B** **)**. In the turquoise (immune negatively related) module, proteins also demonstrated a relatively tight relationship, with the TOP2A (34, 35) (an enzyme that controls and alters the topologic states of DNA during transcription) having the most interactions of 80 (**Figures 3C, D** **)**. Generally, there are strong interactions among hub genes which allowed them to be regulated together.

### The Identification of GW-8510 and Its Anti-Tumor Activity in TNBC

To regulate the abovementioned hub genes globally and increase immune cell infiltration, the changes of hub genes were analyzed by Connectivity Map analysis (CMap), and promising molecules were discovered and shown in **Table 1**. Among these recommended drugs, GW-8510 exhibited the highest enrichment score, which indicated that it may be the best-fitting drug for the investigated purpose. As GW-8510 has little research on its anti-tumor effect in TNBC, we first tested its anti-tumor activity *in vitro*. Human (MDA-MB-231, BT549) and mouse (4T1) breast cancer cell lines were treated with different concentrations of GW-8510. The result showed that 2.5 μM of GW-8510 could decrease the viability of TNBC cells after 24-hour treatment, and higher doses of GW-8510 (5 and 10 μM) would result in more effective inhibition of cancer cells (**Figure S4A**). To further confirm the tumor-suppressive activity of GW-8510, colony formation assays were performed and found that the number of cells was significantly reduced upon 1.25 μM and 0.625 μM of GW-8510 24 h exposure (**Figure S4B**).

**Table 1:** Potential effective small-molecule agents predicted by CMap.

CMap name	n	Enrichment	p	Specificity	Percent non-null
GW-8510	4	0.952	0	0.0663	100
phenoxybenzamine	4	0.925	0.00004	0.1485	100
MS-275	2	0.921	0.01221	0.1255	100
daunorubicin	4	0.895	0.0001	0.0404	100
DL-thiorphan	2	0.893	0.02372	0.0882	100
menadione	2	0.876	0.03068	0.1733	100
rottlerin	3	0.868	0.00427	0.0985	100
blebbistatin	2	0.859	0.03986	0.0732	100
thioguanosine	4	0.849	0.00074	0.0294	100
medrysone	6	0.827	0.00004	0.0079	100

### GW-8510 Induces Pyroptosis *via* Activated Caspase-3 and Cleaved GSDME

**Figure 4:**
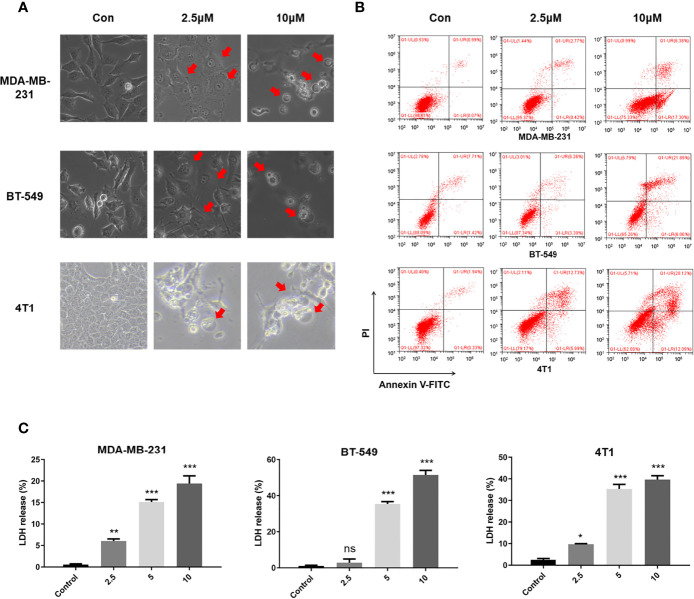
GW-8510 induces pyroptotic cell death in TNBC cells. **(A)** Representative phase-contrast images of GW-8510 treated cells with 0, 2.5, and 10 μM for 24 h. Original magnification, ×400. **(B)** Flow cytometry analysis of GW-8510-treated TNBC cells stained by Annexin V-FITC and PI. **(C)** Release of LDH from TNBC cells treated with indicated concentration of GW-8510 for 24 h. *P < 0.05, **P < 0.01, and ***P < 0.001 were considered statistically significant.

**Figure 5:**
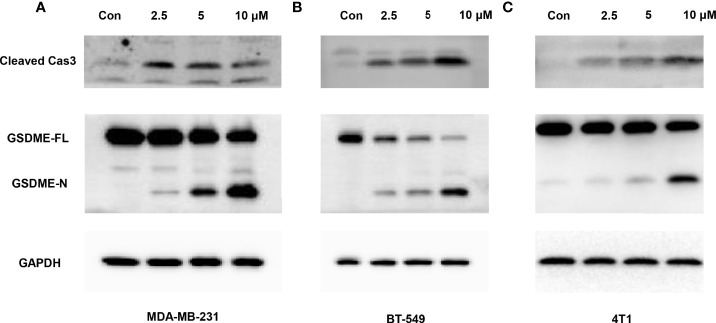
Caspase-3-mediated cleavage of GSDME is involved in GW-8510-induced pyroptosis in TNBC cells. Representative immunoblot analysis of cleaved caspase-3 and N-terminal fragments of GSDME in **(A, B)** human TNBC cells (MDA-MB-231, BT549) cells and **(C)** mouse TNBC cells (4T1) treated with GW-8510 with 0, 2.5, 5, and 10 μM for 24 h. GAPDH was used as an internal control.

When treated with GW-8510, TNBC cells exhibited microscopic features of cell swelling and balloon-like bubbling, which are morphological features of pyroptotic cells (36, 37) (**Figure 4A**). Considering that cancer cell pyroptosis would result in inflammation in the tumor microenvironment and increase immune cell infiltration (38), we further investigated whether GW-8510 could induce pyroptosis in TNBC. Since pyroptotic cells were positive for both Annexin V and PI (37, 39), we ran the flow cytometry analysis and observed an increase in Annexin V+/PI+ cells after **10 μM of GW-8510 treatment for 24 h** (**Figure 4B**). Furthermore, the release of lactate dehydrogenase (LDH) was measured as an indication of pyroptotic cell cytotoxicity in previous studies (40), since pyroptosis could break the plasma membrane integrity and release cytosolic components. The results displayed that GW-8510 treatment significantly increased the LDH release of TNBC cells in a dose-dependent manner (**Figure 4C**). For the next step, we investigated whether caspase-3/GSDME was involved in GW-8510 induced pyroptosis since caspase-3 activation followed by clipping of GSDME within the N terminus plays a major part in switching apoptotic cell death to pyroptotic cell death in various cancers (41, 42). Results showed that GW-8510 treatment elevated the level of N-terminal fragments of GSDME with concomitant cleavage of caspase-3 in a dose-dependent manner (**Figure 5**). Taken together, these data suggest that GW-8510 may significantly induce pyroptosis *via* caspase-3 and GSDME activation.

**Figure 6:**
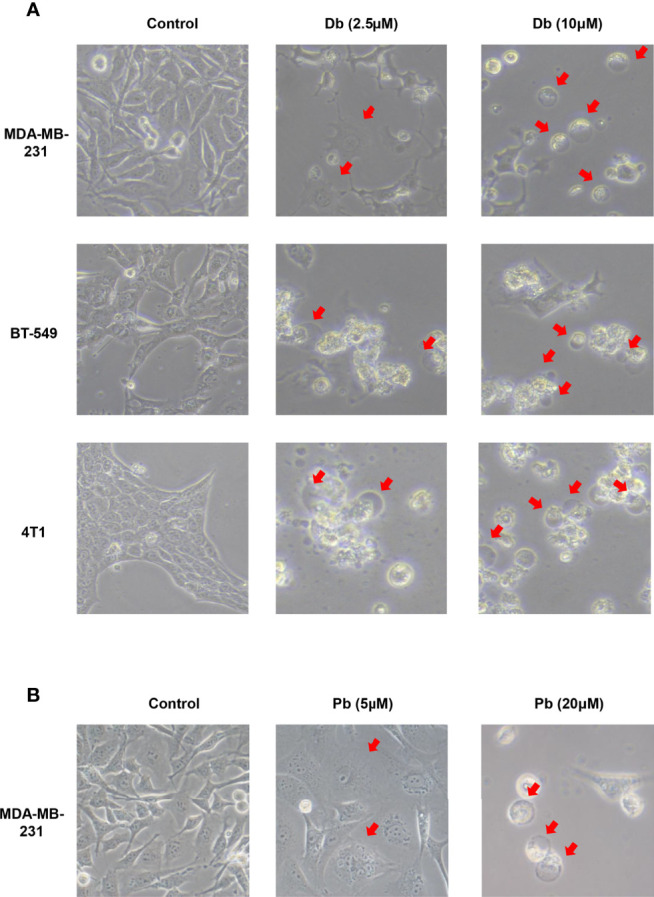
Pyroptotic cell death induced by Dinaciclib and Palbociclib in TNBC cells. Representative phase-contrast images of Dinaciclib **(A)** and Palbociclib **(B)** treated cells with indicated concentration for 24 h. Original magnification, ×400.

**Figure 7:**
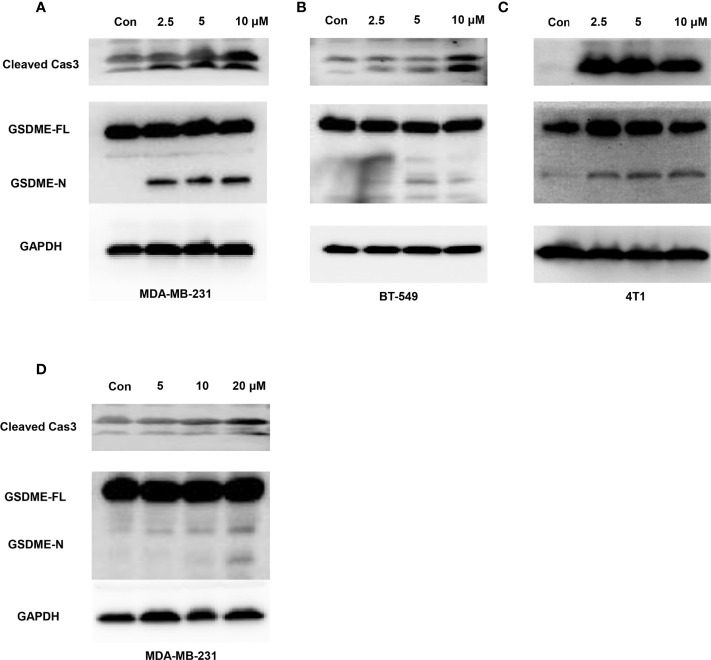
Caspase-3-mediated cleavage of GSDME induced by Dinaciclib and Palbociclib in TNBC cells. Representative immunoblot analysis of cleaved caspase-3 and N-terminal fragments of GSDME in MDA-MB-231 **(A)**, BT549 **(B)**, and 4T1 **(C)** cells treated with Dinaciclib with 0, 2.5, 5, and 10 μM for 24 h. **(D)** Representative immunoblot analysis of cleaved caspase-3 and N-terminal fragments of GSDME in MDA-MB-231 cells treated with Palbociclib with 0, 5, 10, and 20 μM for 24 h. GAPDH was used as an internal control.

Moreover, considering that GW-8510 is an inhibitor of CDK2 and CDK inhibitors have demonstrated promising therapeutic potentials in TNBC, we further investigated the ability to promote pyroptosis in other CDK inhibitors including a broad-acting CDK inhibitor Dinaciclib and a highly selective CDK4/6 inhibitor Palbociclib. As we expected, both of them could induce pyroptosis-specific morphological features such as cell swelling and balloon-like bubbling (**Figure 6**) and increase the cleaved caspase 3 and N-terminal fragments of GSDME (**Figure 7** **)**. Meanwhile, it has been reported that other members of the gasdermin family, like GSDMB, GSDMC also involved in the pyroptosis of cancer cells (36). To find out whether these molecules participated in the CDK inhibitor-induced pyroptosis, the expression of GSDMB and GSDMC was tested by western blot, and no N-terminal fragments were detected for GSDMB and GSDMC after CDK inhibitors treatments (**Figure S5**). Taken together, our *in vitro* studies provided evidence that CDK, inhibitors including GW-8510, Dinaciclib, and Palbociclib, exerted their anti-tumor effect possibility through pyroptosis, which could further ignite an anti-tumor immune response.

### Dinaciclib Induces Pyroptosis of Cancer Cells *In Vivo* and Renders Infiltration of Immune Cells

**Figure 8:**
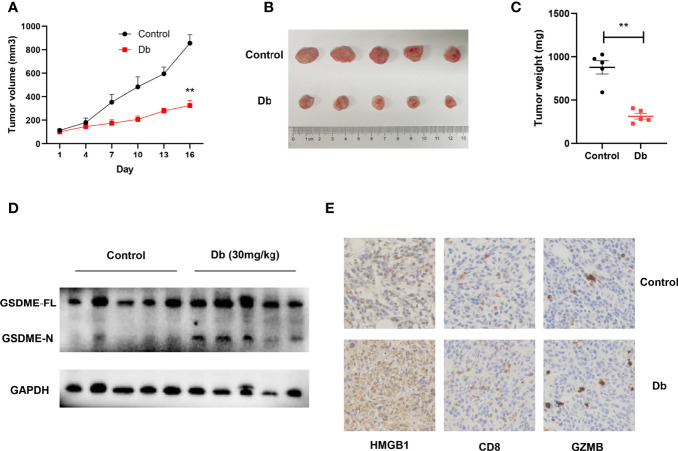
Dinaciclib induces pyroptosis of tumor cells in immune-competent mice models, resulting in increased infiltration of immune cells. **(A–E)** Mice were subcutaneously injected with 4T1 cells. Intraperitoneal injections of Db (30mg/kg and 3 times per week, n = 5) or vehicle (n = 5) lasted for 2 weeks after 4T1 cell inoculation. **(A)** Tumor growth curve. **(B)** Representative image of tumors harvested from mice. **(C)** Tumor weight of mice. **(D)** Western blots showing indicated protein changes in tumor tissues after treatment with Dinaciclib or vehicle in 4T1 xenografts. **(E)** Representative histologic sections of xenografts from tumors of 4T1 were staining with HMGB1 (left), CD8 (middle), and granzyme B (right). **, p<0.01 calculated by the student t test.

*In vivo* studies were further conducted to better demonstrate the pyroptosis-inducing and immune-evoking ability of CDK inhibitors. Dinaciclib was chosen for subsequent experiments considering its accessibility and potential in clinical applications (43). Mouse breast cancer cells (4T1 cell line) were injected subcutaneously in immune-competent mice under general anesthesia. The treatment group were administrated with Dinaciclib (30mg/kg, 3 times per week, i.p), and mice in the control group were treated by the same volume of vehicle. Consisting with *in vitro* results, Dinaciclib treatment significantly restricted the growth of xenograft tumors (**Figure 8A**). At the time of sacrifice, the tumor volume and weight were significantly lower in the Dinaciclib group than the controls (**Figures 8B, C**). Furthermore, western blots for tumor samples showed more cleaved GSDME-N after Dinaciclib treatment (**Figure 8D**). In the meantime, HMGB1, as one indicator of immunogenic cell death, was much higher in tumor samples collected from the Dinaciclib group (**Figure 8E**). Consistently, CD8 T cells and granzyme B were also increased after Dinaciclib treatment (**Figure 8E**).

## Discussion

In this study, hub genes with the potential to increase immune cell infiltration and enhance immunotherapy efficiency in TNBC were identified using bioinformatics analysis. What’s more, GW-8510, a CDK2 inhibitor, was recommended by the CMap database to achieve the global modification of hub genes. *In vitro* and *in vivo* studies were performed and revealed the anti-tumor effect of GW-8510 and other CDK inhibitors. Meanwhile, GSDME-mediated pyroptotic phenotype was validated in these CDK inhibitors, highlighting their immune evoking abilities and the possibilities of combining immunotherapies with CDK inhibitors in TNBC.

According to the gene enrichment analysis, hub genes exhibited high involvements in immune-related biological processes, which could also be validated by the central role of serval vital immune molecules (CD45, CD8, LCK, and CD3) in the PPI network. The immunoregulatory function of hub genes was consisted with our study objectives and supported the validity of our *in silico* analysis. Meanwhile. the strong associations among hub genes provided reassurance for modulating the overall hub gene network and added significance for the CMap predicted drugs (44, 45). Among the top 10 fittest drugs revealed by CMap, apart from GW-8510, MS-275 (also known as Entinostat) was previously reported to help reprogram the tumor’s innate and adaptive immune landscape and induce an anti-tumor response in multiple human tumor types (46–50), also indicating the good reliability of our research strategies.

CDK inhibitors, highlighted in this study, emerged as novel target therapies in breast cancer. The aberrant expression of cyclin-dependent kinases were common features among various malignancies including breast cancer (51, 52). CDK4/6 inhibitors such as palbociclib, ribociclib, and abemaciclib could induce cell cycle arrest and enable better control over tumor progression (53). Thus, CDK4/6 inhibitors achieved broad clinical applications in the current breast cancer treatments (54, 55). However, intrinsic or acquired resistance to clinically approved CDK4/6 inhibitors have emerged as a major obstacle that hinders their utility in breast cancers (56). Other CDK inhibitors including CDK2 inhibitors were therefore introduced (57). Previous studies reported that GW-8510 could suppress lung cancer cell proliferation and re-sensitize them to gemcitabine through autophagy induction (58). And its role in the anti-tumor activity and immune modulation in TNBC was revealed for the first time in this article, as well as the pro-pyroptosis effect of GW-8510 and other CDK inhibitors.

The role of CDK inhibitors in anti-cancer immune and its potential combined therapy with immune checkpoint blockers have been noticed in previous studies (59–61). Recently, researchers recognized that tumor regression mediated by CDK4/6 inhibition is partially dependent on the presence of cytotoxic T cells (61). Other CDK inhibitors such as CDK12/13 inhibitors were also reported to induce immune death in different cancers. In clinical trials, a Phase Ib trial (NCT02779751) aimed to assess the safety and antitumor activity of abemaciclib plus pembrolizumab in patients with endocrine-resistant, metastatic ER+ breast cancers reported an overall response rate (ORR) of 29%, a high disease control rate of 82%, and a clinical benefit rate of 46%, along with higher durations of PFS (8.9 months) and OS (26.3 months) compared to abemaciclib monotherapy. Another CDK4/6 inhibitor-based immunotherapy combination (palbociclib plus pembrolizumab) in a Phase II study in postmenopausal patients with metastatic ER+ breast cancer patients (NCT02778685) also demonstrated a prolonged median follow-up time of 13.7 months and increased partial response rate of 42.1% (62).

Despite the progress, underlying mechanisms for the immune modulation ability of CDK inhibitors remained poorly investigated. In this study, our results indicated that pyroptosis can be induced by different CDK inhibitors, which might provide serval new insights into this question. Pyroptosis is a lytic pro-inflammatory type of cell death depending on the formation of gasdermin pore on the plasma membrane and pore-induced membrane lysis (63). Pyroptotic cells would release “find me” and “eat me” molecular signals and thus boost antitumor immunity. Among gasdermins, GSDME was a potent executor that could be cleaved by the apoptotic caspase-3 and induce robust pyroptosis in different types of cancer cells (64–66). In this study, TNBC cancer cells treatment with GW-8510 and two other CDK inhibitors all showed an increased level of cleaved caspase-3 and N-terminal fragments of GSDME, which is an indication of GSDME-mediated pyroptosis.

There are several limitations to this study. First, the role of hub proteins obtained from bioinformatic analysis were not verified by clinical samples, which should be further investigated in the future. Second, only GEO dataset were included in current analysis, which may limited the broader application of study results. Besides, whether the ability to induce pyroptosis of CDK inhibitors is specific to cancer cells or to both cancer cells and immune cells should be further estimated.

In conclusion, hub genes related to immune infiltrations were identified in TNBC. A CDK 2 inhibitor, GW-8510, was predicted to be able to improve anti-tumor immunity by globally modulating these genes. The *in vitro* and *in vivo* studies verified the potent anti-tumor activity of CDK inhibitors. More importantly, these CDK inhibitors could trigger pyroptosis *via* the activation of caspase 3 and GSDME, which could be the mechanism for their potential in boosting immune and enhancing immunotherapy efficiency.

## Data Availability Statement

The original contributions presented in the study are included in the article/**Supplementary Material** Further inquiries can be directed to the corresponding authors.

## Ethics Statement

The animal study was reviewed and approved by the Care and Treatment of Laboratory Animals of Tongji Hospital and approved by the Ethics Committees of Tongji Hospital.

## Author Contributions

QG, YX, and XL designed the experiments and supervised the study. TX, ZW, and JL collected, analyzed, and interpreted the data. GW, DZ, and YD participated in revising the manuscript. All authors have read and approved the final manuscript.

## Funding

This work was supported by the National Natural Science Foundation of China (81902933, 81802676).

## Conflict of Interest

The authors declare that the research was conducted in the absence of any commercial or financial relationships that could be construed as a potential conflict of interest.

## Publisher’s Note

All claims expressed in this article are solely those of the authors and do not necessarily represent those of their affiliated organizations, or those of the publisher, the editors and the reviewers. Any product that may be evaluated in this article, or claim that may be made by its manufacturer, is not guaranteed or endorsed by the publisher.
