# Supplementary material for: Cyclin-Dependent Kinase Inhibitors Function as Potential Immune Regulators via Inducing Pyroptosis in Triple Negative Breast Cancer
Source: Front Oncol. 2022 Jun 8;12:820696. doi: 10.3389/fonc.2022.820696 (PMC9213695; doi:10.3389/fonc.2022.820696)
Supplement: Supplementary file 1 [file DataSheet_1.docx]

Supplementary Material

# Supplementary Figures


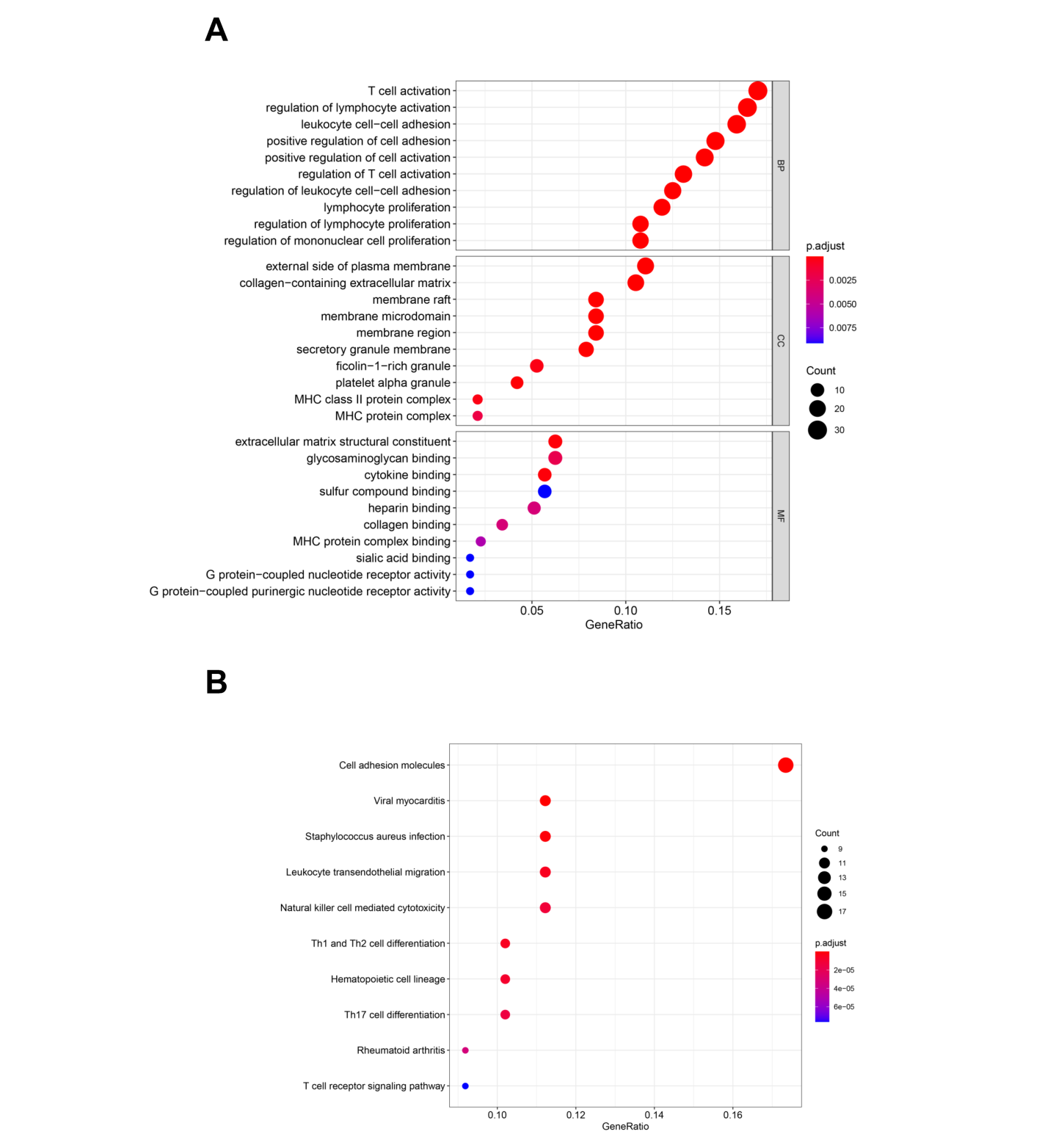


**Supplement Figure 1.** GO and KEGG enrichment analysis for immune positive-related hub genes.

(A) GO enrichment; (B) KEGG pathway enrichment.


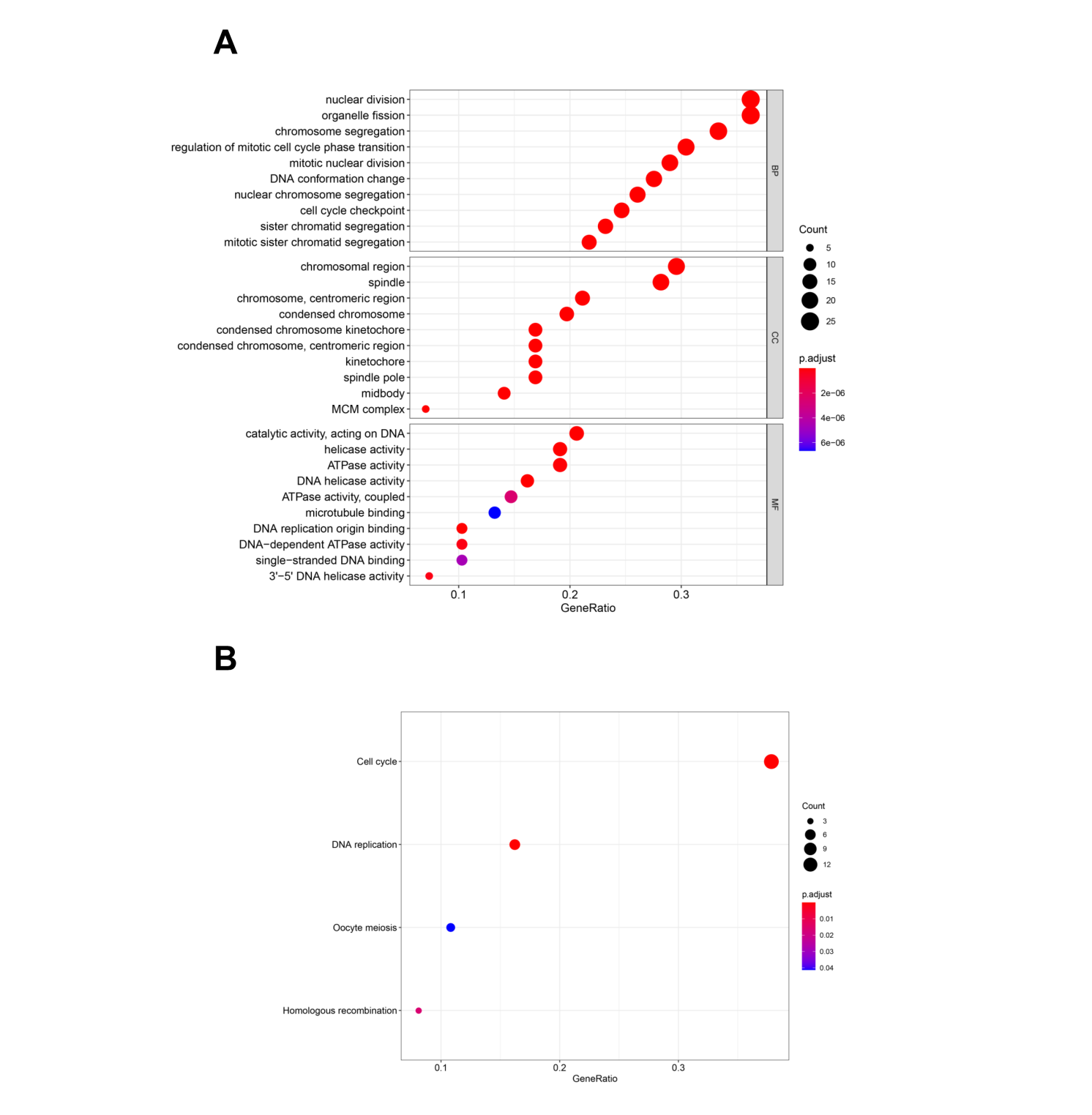


**Supplement Figure 2.** GO and KEGG enrichment analysis for immune negative-related hub genes.

(A) GO enrichment; (B) KEGG pathway enrichment.


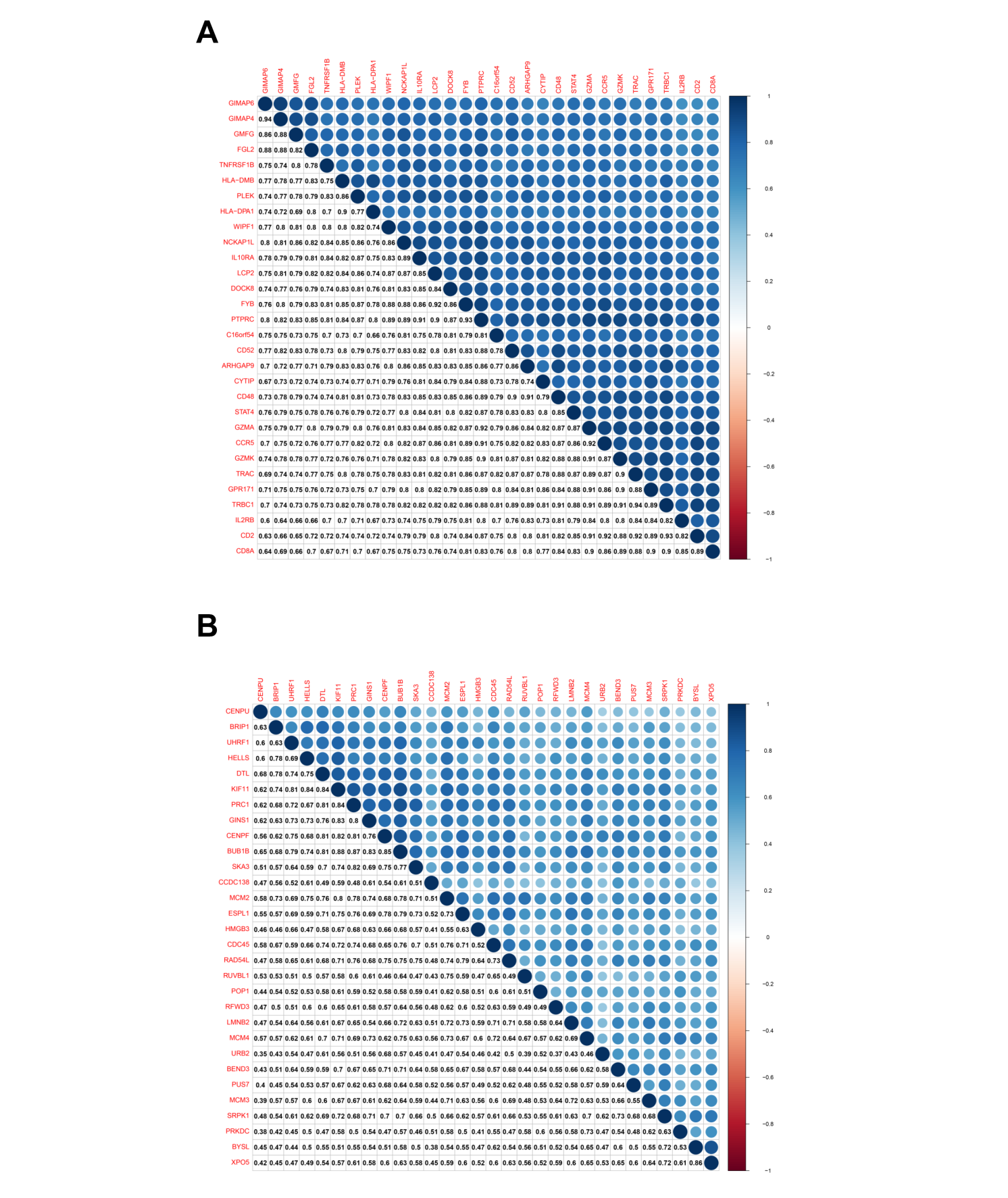


**Supplement Figure 3.** The correlation between hub genes. The correlation plot between hub genes in the immune positive- (A) and negative- (B) related modules. The upper-right part of the figures is the degree of correlation presented by different colors, and the lower-left part is the correlation value.

**
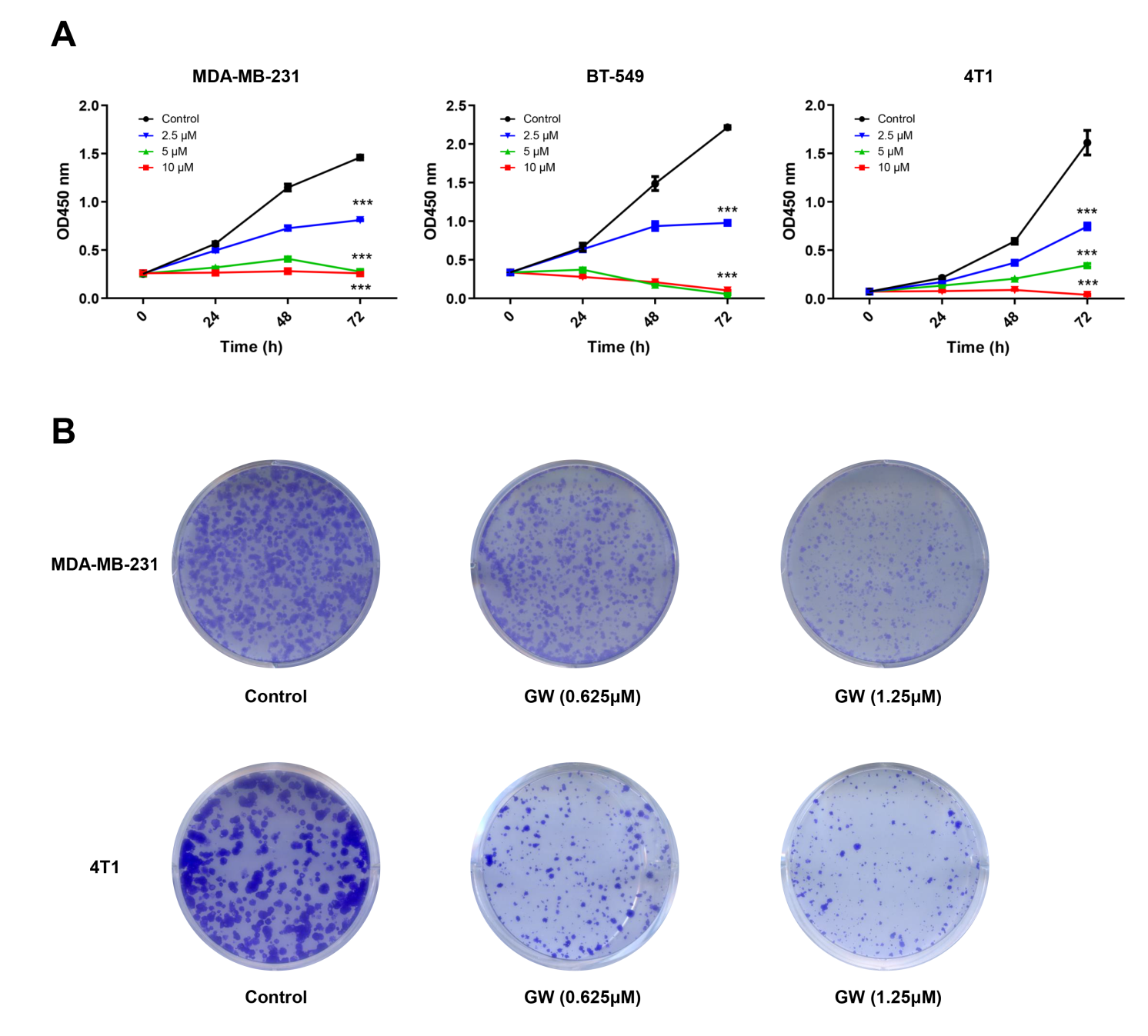
**

**Supplement Figure 4.** GW-8510 inhibits TNBC cell growthin vitro.(A) Breast cancer cell lines MDA-MB-231, BT549, and 4T1 were treated with a gradient concentration of GW-8510 for 24 h, 48 h, or 72 h. Relative cell viability was analyzed by CCK-8 assay. (B) Representative images of colony formation assay were measured after MDA-MB-231, and 4T1 colony initiation being treated to different concentration of GW-8510 for 24 h.


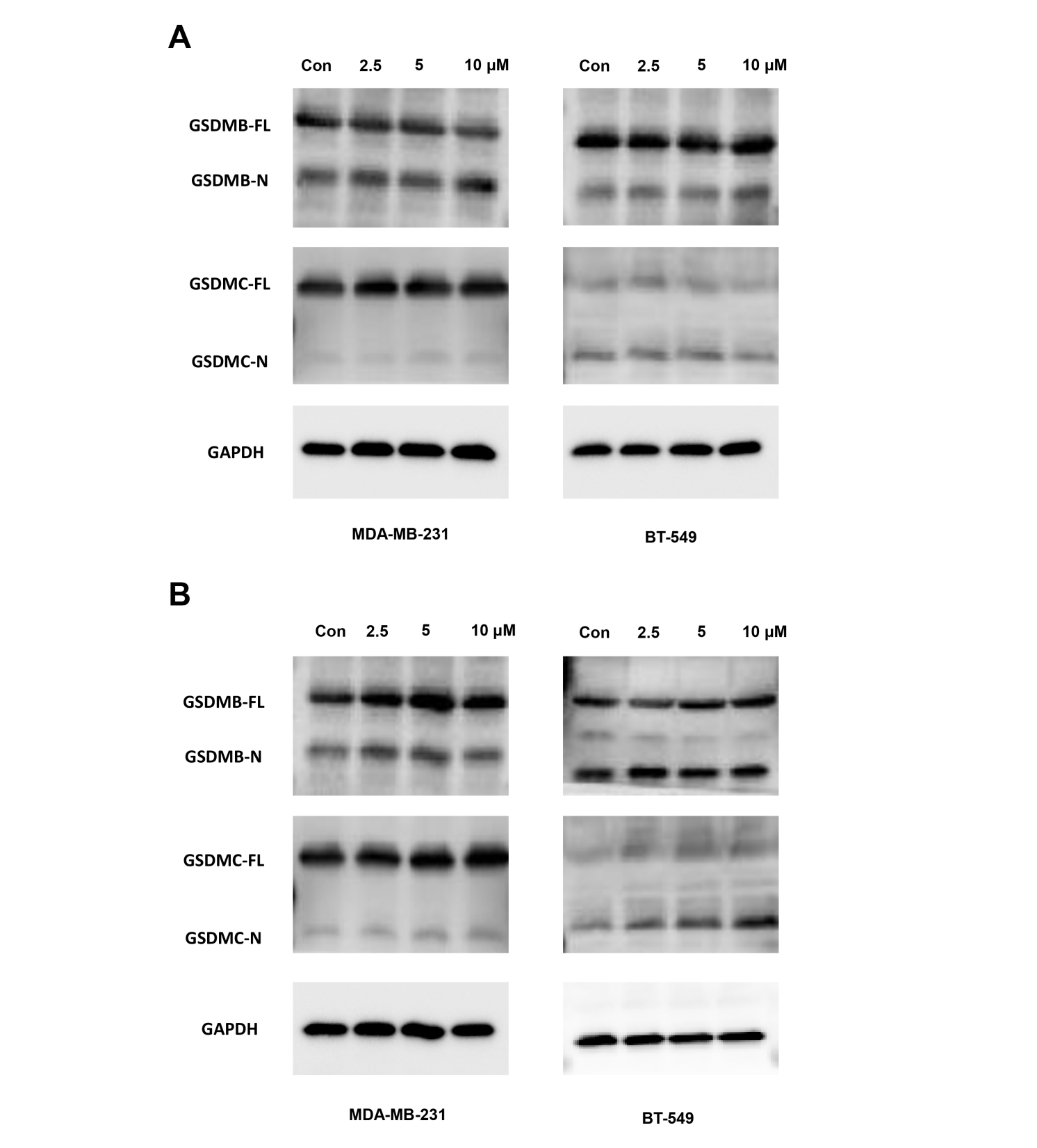


**Supplement Figure 5.** GSDMB and GSDMC are not involved in GW-8510 and Dinaciclib-induced pyroptosis in TNBC cells. Representative immunoblot analysis of N-terminal fragments of GSDMB and GSDMC in MDA-MB-231 and BT549 cells treated with GW-8510 (A) and Dinaciclib (B) with 0, 2.5, 5, and 10 μM for 24 h. GAPDH was used as an internal control.
